# Dimethylfumarate Attenuates Renal Fibrosis via NF-E2-Related Factor 2-Mediated Inhibition of Transforming Growth Factor-β/Smad Signaling

**DOI:** 10.1371/journal.pone.0045870

**Published:** 2012-10-08

**Authors:** Chang Joo Oh, Joon-Young Kim, Young-Keun Choi, Han-Jong Kim, Ji-Yun Jeong, Kwi-Hyun Bae, Keun-Gyu Park, In-Kyu Lee

**Affiliations:** 1 Departments of Internal Medicine, Research Institute of Aging and Metabolism, WCU Program, Kyungpook National University School of Medicine, Daegu, Republic of Korea; 2 GIST College, Gwangju Institute of Science and Technology, Gwangju, Republic of Korea; 3 Research Institute of Clinical Medicine, Chonnam National University Hwasun Hospital, Hwasun, Republic of Korea; UAE University, United Arab Emirates

## Abstract

TGF-β plays a key role in the development of renal fibrosis. Suppressing the TGF-β signaling pathway is a possible therapeutic approach for preventing this disease, and reports have suggested that Nrf2 protects against renal fibrosis by inhibiting TGF-β signaling. This study examines whether dimethylfumarate (DMF), which stimulates Nrf2, prevents renal fibrosis via the Nrf2-mediated suppression of TGF-β signaling. Results showed that DMF increased nuclear levels of Nrf2, and both DMF and adenovirus-mediated overexpression of Nrf2 (Ad-Nrf2) decreased PAI-1, alpha-smooth muscle actin (α-SMA), fibronectin and type 1 collagen expression in TGF-β-treated rat mesangial cells (RMCs) and renal fibroblast cells (NRK-49F). Additionally, DMF and Ad-Nrf2 repressed TGF-β-stimulated Smad3 activity by inhibiting Smad3 phosphorylation, which was restored by siRNA-mediated knockdown of Nrf2 expression. However, downregulation of the antioxidant response element (ARE)-driven Nrf2 target genes such as NQO1, HO-1 and glutathione S-transferase (GST) did not reverse the inhibitory effect of DMF on TGF-β-induced upregulation of profibrotic genes or extracellular matrix proteins, suggesting an ARE-independent anti-fibrotic activity of DMF. Finally, DMF suppressed unilateral ureteral obstruction (UUO)-induced renal fibrosis and α-SMA, fibronectin and type 1 collagen expression in the obstructed kidneys from UUO mice, along with increased and decreased expression of Nrf2 and phospho-Smad3, respectively. In summary, DMF attenuated renal fibrosis via the Nrf2-mediated inhibition of TGF-β/Smad3 signaling in an ARE-independent manner, suggesting that DMF could be used to treat renal fibrosis.

## Introduction

The progressive accumulation of extracellular matrix (ECM) components in renal parenchyma leading to end stage renal disease is a characteristic feature of chronic kidney diseases [1], [2]. A number of profibrotic growth factors, including transforming growth factor-beta (TGF-β), connective tissue growth factor, platelet-derived growth factor (PDGF) and fibroblast growth factor (FGF), have been implicated in the pathogenesis of ECM accumulation [3]–[5]. Several lines of evidence from both animal and human studies have suggested a critical role for TGF-β in the development of renal fibrosis, and this evidence is supported by studies showing that TGF-β not only stimulates matrix protein generation but also inhibits matrix protein removal [6]–[8]. The upregulation of TGF-β expression has been demonstrated in a variety of renal diseases, including obstructive nephropathy. Increased TGF-β expression in the obstructed kidney stimulated genes involved in ECM protein accumulation including type 1 collagen and fibronectin [9]. Additionally, TGF-β stabilizes ECM proteins by stimulating the expression of plasminogen activator inhibitor 1 (PAI-1). Thus, the inhibition of TGF-β signaling has been included in several therapeutic approaches for preventing renal fibrosis [2], [10].

Dimethylfumarate (DMF) is an orally bioavailable fumaric acid ester currently used for the treatment of psoriasis [11]–[13]. In addition, DMF attenuates multiple sclerosis, an immune-mediated inflammatory disease that attacks myelinated axons in the central nervous system [14]–[18] and inhibits tumor cell invasion [19]–[21]. DMF has also been shown to reduce airway smooth muscle cell proliferation through the induction of heme oxygenase (HO)-1 [22]. Recent studies have shown that DMF increases the expression of NF-E2-related factor 2 (Nrf2), which is repressed by binding to the inhibitor Keap1 in the cytoplasm [17], [23], [24]. Keap1, an Nrf2 inhibitor, consists of three protein domains, one of which, the intervening region (IVR) contains several thiol groups and DMF interacts with these thiol groups to induce a conformational change in Keap1. As a result of this conformational change, Nrf2 dissociates from Keap1 and enters the nucleus, where it induces the expression of various antioxidant/detoxification enzymes [23], [25]–[27].

Nrf2 is a basic leucine-zipper (bZip) transcription factor that protects a variety of tissues and cells against oxidative and electrophilic stress through antioxidant response element (ARE)-mediated induction of diverse phase II detoxification and antioxidant enzymes, including NAD(P)H quinone oxidoreductase 1 (NQO1) and heme oxygenase-1 (HO-1) [26], [28]. Recently several studies demonstrated that Nrf2 protects against streptozotocin-induced diabetic nephropathy [29] and mitigates cisplatin-induced nephrotoxicity through the induction of antioxidant enzymes [30]. In addition, Nrf2 attenuates cyclosporin A-induced epithelial-mesenchymal transition in renal fibrosis via the induction of HO-1 [31] and inhibits inflammation and sclerosis in focal segmental glomerulosclerosis in mice [32]. Therefore, chemicals that modulate the activity of Nrf2 and regulate the TGF-β signaling pathway represent potential therapeutic agents for the treatment of renal fibrosis. Until now, protective effect of DMF, one of the Nrf2 activators, against renal fibrosis has not been investigated.

In this study, we examined the potential effects of DMF on TGF-β-stimulated ECM production *in vitro* and unilateral urethral obstruction (UUO)-induced renal fibrosis *in vivo*, and elucidated its underlying molecular mechanisms which are associated with Nrf2-mediated inhibition of the TGF-β/Smad3 signaling pathway.

## Results

### DMF inhibits TGF-β-stimulated PAI-1, α-SMA, fibronectin and type I collagen expression

**Figure 1:**
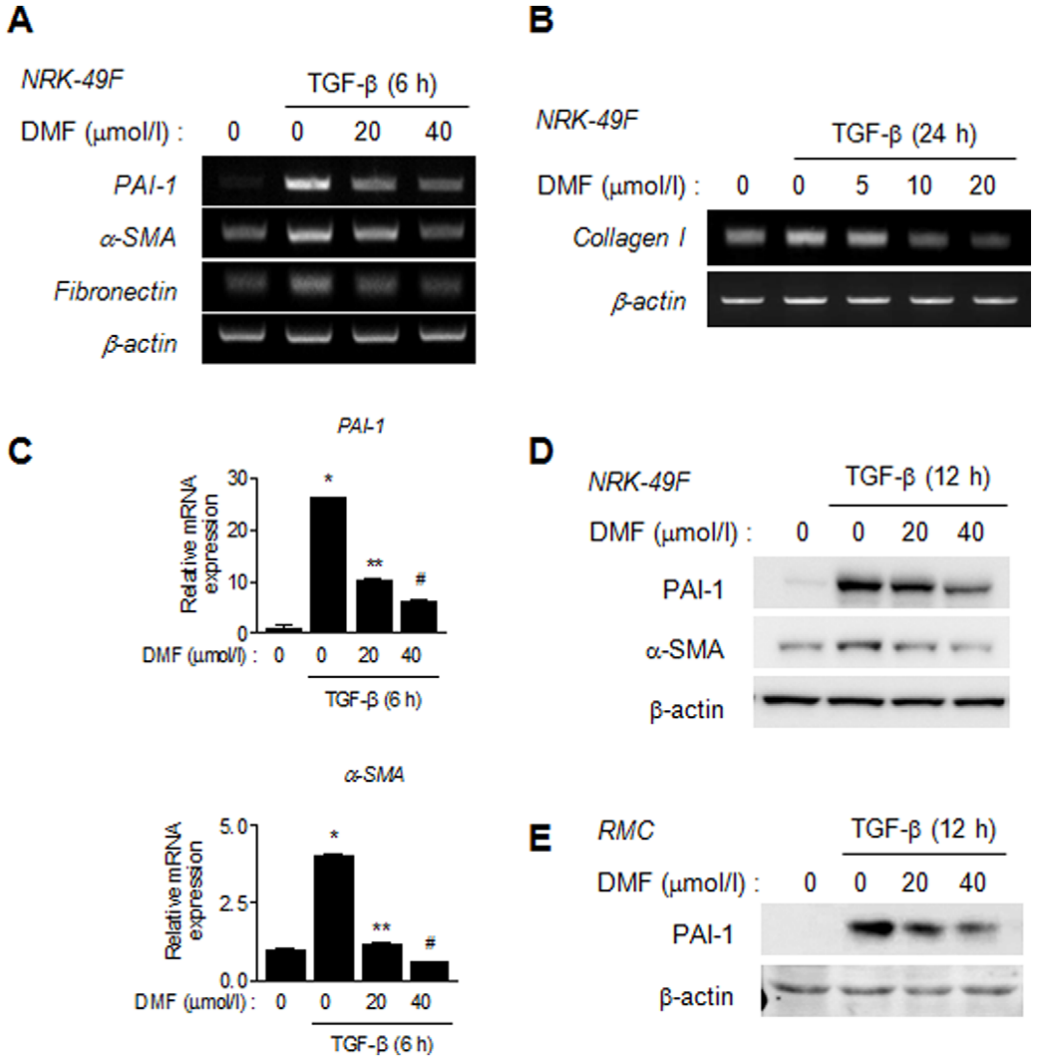
DMF inhibits TGF-β-stimulated PAI-1, α-SMA, fibronectin and type 1 collagen expression. (A and B) Representative semi-quantitative RT-PCR analysis of the effects of DMF on PAI-1, α-SMA, fibronectin and type 1 collagen expression in TGF-β-stimulated NRK-49F cells. NRK-49F cells were starved for 12 h, treated with DMF for 1 h, and then stimulated with TGF-β (2 ng/ml) for 6 h (A) or 24 h (B). (C) Representative real-time RT-PCR analysis of the effects of DMF on TGF-β-stimulated PAI-1 and α-SMA mRNA expression. NRK-49F cells were treated with DMF for 1 h and then stimulated with TGF-β (2 ng/ml) for 6 h. Cells were harvested for real-time PCR analysis, and data represent the means ±SE of three independent measurements. ^*^ *P*<0.01 vs. control; ^**^ *P*<0.01, ^#^ *P*<0.001 vs. TGF-β stimulation (upper panel); ^*^ *P*<0.001 vs. control; ^**^ *P*<0.01, ^#^ *P*<0.001 vs. TGF-β stimulation (lower panel). (D and E) Representative Western blot analysis of the effect of DMF on PAI-1 expression in TGF-β-stimulated NRK-49F (D) and RMC (E) cells. Cells were starved for 12 h and pretreated with DMF for 1 h and then stimulated with TGF-β (2 ng/ml) for 12 h.

Firstly, the effect of DMF on TGF-β-stimulated profibrotic genes and on ECM protein expression was examined in a normal rat renal fibroblast cell line (NRK-49F). As shown in Figure 1A–D, DMF inhibited TGF-β-stimulated PAI-1, α-SMA and fibronectin mRNA and protein expression in a dose-dependent manner. The expression of type I collagen mRNA and protein had not increased at 6 h after treatment with TGF-β (data not shown); however, its expression was induced at 24 h after TGF-β treatment, after which time DMF was seen to inhibit TGF-β-stimulated type I collagen expression in a dose-dependent manner (Figure 1B). The inhibitory effect of DMF on TGF-β-stimulated PAI-1 protein expression was confirmed further in RMCs, a rat mesangial cell line (Figure 1E). These data demonstrated that DMF inhibits TGF-β-stimulated profibrotic genes and ECM protein expression in rat kidney cell lines.

### DMF inhibits the TGF-β/Smad signaling pathway

**Figure 2:**
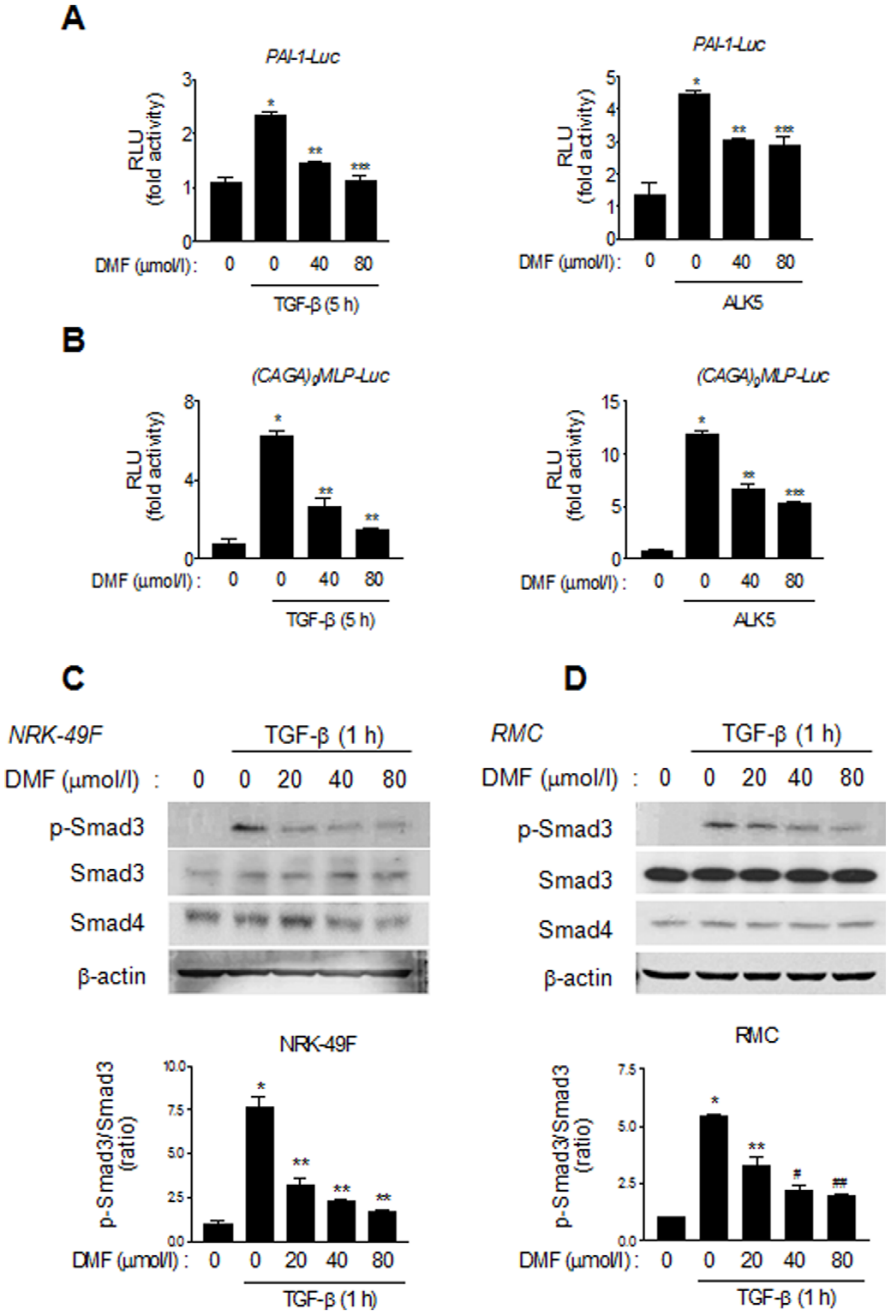
DMF inhibits the TGF-β/Smad3 signaling pathway. (A and B) Effects of DMF on TGF-β-stimulated PAI-1-Luc and (CAGA)_9_MLP-Luc reporter constructs. AD-293 cells were transfected with PAI-1-Luc (A) or (CAGA)_9_MLP-Luc (B) plasmids with or without ALK5, a constitutively active TGF-β type I receptor, for 24 h. Cells were starved for 12 h and stimulated with TGF-β (2 ng/ml) for 5 h. Data represent the means ±SEM of three independent measurements. (A) ^*^ *P*<0.001 vs. reporter alone; ^**^ *P*<0.001, ^***^ *P*<0.01 vs. TGF-β stimulation (left panel). ^*^ *P*<0.05 vs. reporter alone; ^**^ *P*<0.01, ^***^ *P*<0.05 vs. ALK5 stimulation (right panel). (B) ^*^ *P*<0.01 vs. reporter alone; ^**^ *P*<0.01 vs. TGF-β stimulation (left panel). ^*^ *P*<0.01 vs. reporter alone; ^**^ *P*<0.05, ^***^ *P*<0.01 vs. ALK5 stimulation (right panel). (C and D) Representative Western blot analysis of the effect of DMF on TGF-β-stimulated Smad3 phosphorylation. Starved NRK-49F (C) and RMC (D) cells were pretreated with DMF for 1 h and then stimulated with TGF-β (2 ng/ml) for 1 h. Quantitative analysis of p-Smad3 to total Smad3 ratio. Data in bar graph are the mean ± SE of three independent measurements. (C) ^*^ *P*<0.01 vs. control, ^**^ *P*<0.05 vs. TGF-β stimulation. (D) ^*^ *P*<0.001 vs. control, ^**^ *P*<0.05, ^#^ *P*<0.01, *^##^P*<0.001 vs. TGF-β stimulation.

To determine whether DMF represses profibrotic genes and ECM protein expression through the inhibition of the TGF-β-activated Smad signaling pathway, we examined the effect of DMF on a luciferase reporter construct carrying the PAI-1 promoter (PAI-1-Luc), which contains three binding sites for Smad3. Its effect on (CAGA)_9_MLP-Luc promoter activity, a reporter construct that contains nine copies of the Smad3 binding site, was also investigated. As shown in Figure 2A and B, DMF inhibited TGF-β-stimulated PAI-1 promoter activity in AD-293 cells, a derivative of the commonly used HEK-293 human embryonic kidney cell line. DMF also inhibited the activity of (CAGA)_9_MLP-Luc promoter stimulated by either TGF-β or a constitutively active TGF-β type I receptor (ALK5).

Because phosphorylation of Smad3 is a critical step in ensuring the proper TGF-β signaling [33], we evaluated the effect of DMF on the phosphorylation of Smad3 (p-Smad3). As shown in Figure 2C–D, DMF treatment inhibited TGF-β-stimulated Smad3 phosphorylation in NRK-49F (Figure 2C) and RMC cells (Figure 2D). These data indicate that DMF negatively affects TGF-β-mediated transcription via the inhibition of Smad3 phosphorylation.

### DMF-activated Nrf2 protein suppresses the TGF-β-stimulated expression of profibrotic genes

**Figure 3:**
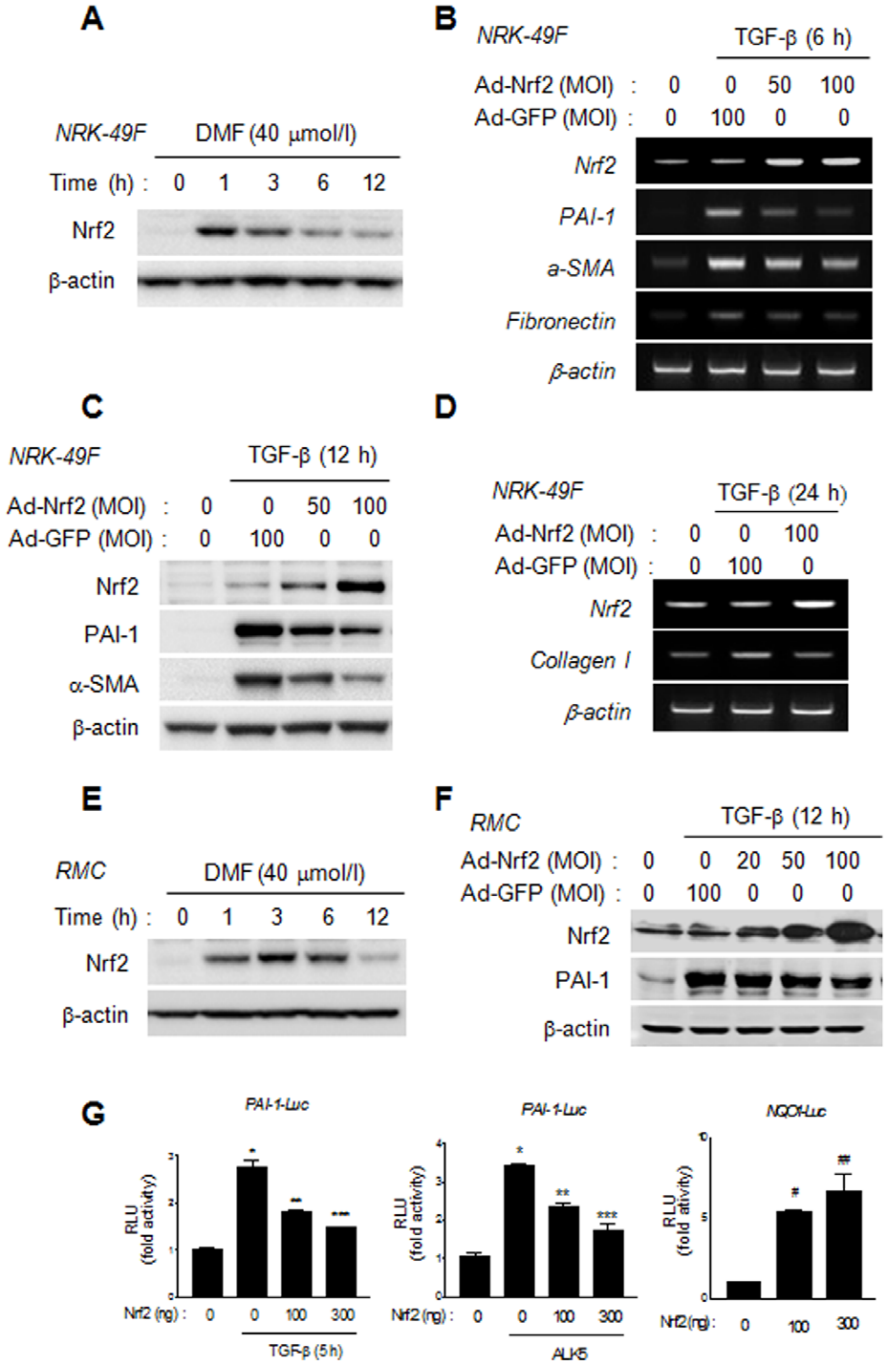
Nrf2 inhibits TGF-β-stimulated PAI-1, α-SMA, fibronectin and type I collagen expression. (A) Representative Western blot analysis of the effect of DMF on Nrf2 expression in NRK-49F cells. Serum-starved cells were treated with DMF (40 µmol/l) for the times indicated and then were harvested for Western blot analysis. (B and D) Representative semi-quantitative RT-PCR analysis of the effects of Ad-Nrf2 on PAI-1, α-SMA, fibronectin and type I collagen expression in TGF-β-stimulated NRK-49F cells. Cells were starved for 24 h after infection with Ad-Nrf2, and then were stimulated with TGF-β (2 ng/ml) for 6 h (B) or 24 h (D). (C) Representative Western blot analysis of the effects of Ad-Nrf2 on PAI-1 and α-SMA and in TGF-β-stimulated NRK-49F cells. Cells, starved for 24 h after infection with Ad-Nrf2, were stimulated with TGF-β (2 ng/ml) for 12 h. (D) Representative semi-quantitative RT-PCR analysis of the effects of Ad-Nrf2 on type I collagen expression in TGF-β-stimulated NRK-49F cells. Cells were starved for 24 h after infection with Ad-Nrf2 and then were stimulated with TGF-β (2 ng/ml) for 24 h. (E) Representative Western blot analysis of the effect of DMF on Nrf2 expression in RMC cells. (F) Representative Western blot analysis of the effect of Ad-Nrf2 on PAI-1 expression in TGF-β-stimulated RMCs. (G) The effect of Nrf2 on TGF-β- or ALK5-stimulated PAI-1-Luc reporters in AD-293 cells. AD-293 cells were cotransfected with PAI-1-Luc plasmids and Nrf2-expression plasmids with or without an ALK5 expression plasmid for 24 h, and then serum starved for 12 h before stimulation with TGF-β for 5 h. Data represent the means ±SEM of three independent measurements. ^*^ *P*<0.01 vs. reporter alone; ^**^ *P*<0.05, ^***^ *P*<0.01 vs. TGF-β stimulation; and ^#^ *P*<0.001, ^##^ *P*<0.05 vs. control.

Since DMF is a well-known activator of the transcription factor Nrf2, which has been implicated in the pathogenesis of renal fibrosis, we investigated whether Nrf2 mediates the suppression of DMF on TGF-β-stimulated profibrotic genes and ECM protein expression. As expected, treatment with DMF resulted in a rapid increase in the protein expression of Nrf2 (Figure 3A). This increased expression of Nrf2 was maintained up to 24 h after co-treatment of DMF with TGF-β in NRK-49F cells (Figure S1), although its expression levels gradually decreased from 1 h after treatment of DMF. DMF also induced the nuclear accumulation of Nrf2 in a dose dependent manner (Figure S2). Because the p62-mediated stabilization of Nrf2 has recently been suggested as an antioxidant-independent mechanism for Nrf2 activation [34], we investigated the effect of DMF on p62 expression and the involvement of p62 in DMF-induced Nrf2 expression. Unlike rapid induction of Nrf2 expression by DMF, p62 expression was augmented at 6 h and further enhanced at 12 h after DMF treatment (Figure S3A), suggesting that DMF increases Nrf2 expression by a p62-independent mechanism. Consistently, a small interfering RNA (siRNA) against p62 (p62-siRNA) had little effect on DMF-increased Nrf2 expression, while it significantly diminished both basal and DMF-induced p62 expression in NRK-49F cells (Figure S3B). Moreover, down-regulation of p62 expression in AD-293 cells did not reverse the inhibitory effects of DMF on (CAGA)_9_MLP-Luc activity and profibrotic gene expression stimulated by TGF-β (Figure S3C–E).

Next, we examined whether Nrf2 suppresses TGF-β-stimulated ECM expression. The results showed that adenovirus-mediated overexpression of Nrf2 (Ad-Nrf2) decreased PAI-1, α-SMA and fibronectin mRNA and protein expression in NRK-49F cells (Figure 3B and C). Ad-Nrf2 also decreased type I collagen mRNA expression at 24 h after TGF-β treatment (Figure 3D). Additionally, we confirmed that DMF causes a rapid increase in expression of the Nrf2 protein in RMC cells (Figure 3E) and that Ad-Nrf2 inhibits TGF-β-stimulated ECM expression in RMCs (Figure 3F). Moreover, transient transfection with Nrf2 decreased PAI-1 promoter activities stimulated by either TGF-β treatment or ALK5 cotransfection in a dose-dependent manner (Figure 3G). In contrast, promoter activity of NQO1, a well-known target gene of Nrf2 was markedly activated by transfection with Nrf2. These data demonstrate that Nrf2 activated by DMF suppresses the TGF-β-induced expression of profibrotic genes.

### Nrf2 inhibits the TGF-β/Smad signaling pathway

**Figure 4:**
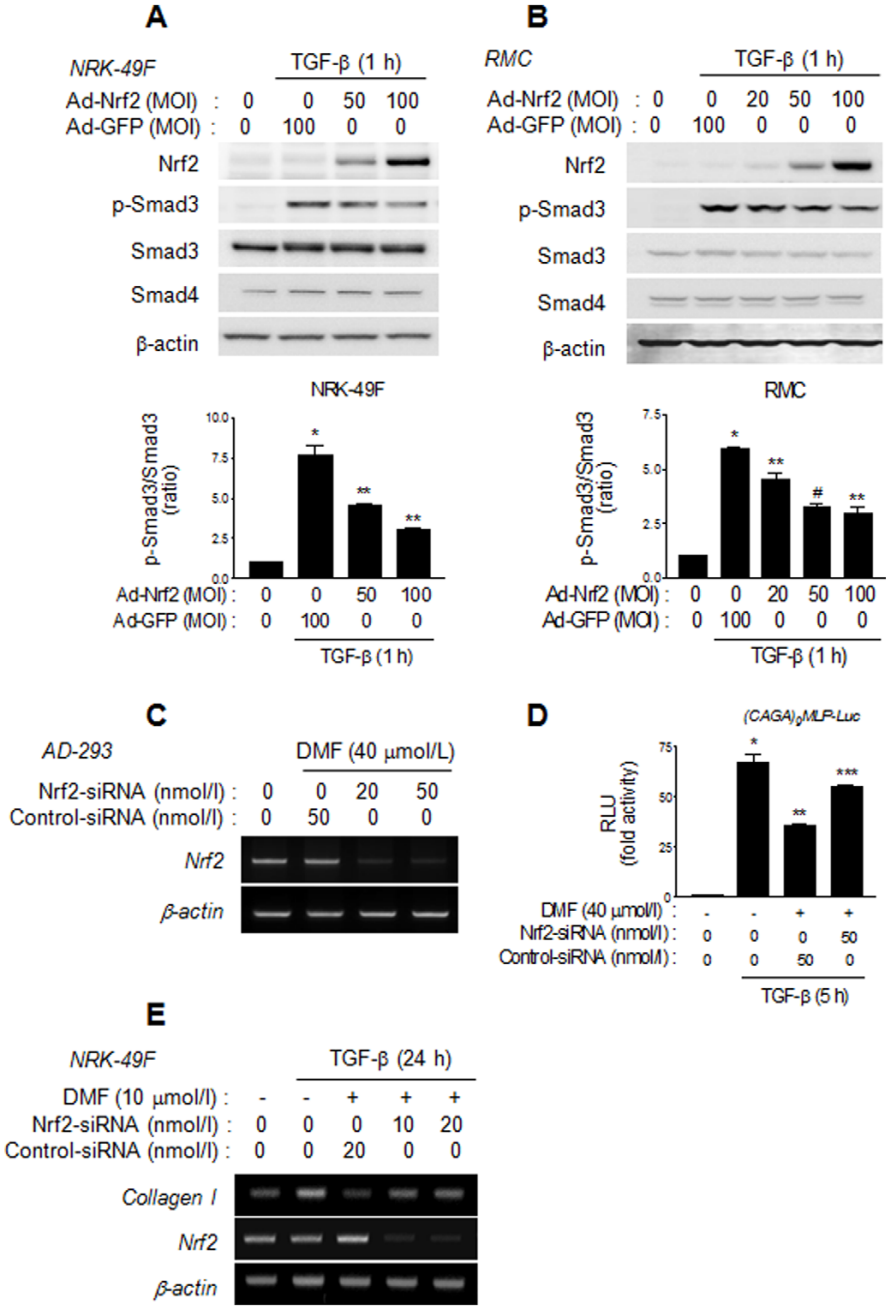
Nrf2 inhibits the TGF-β/Smad3 signaling pathway. (A and B) Representative Western blot analysis of the effect of Ad-Nrf2 on Smad3 phosphorylation in TGF-β-stimulated NRK-49F (A) and RMC (B) cells. Cells were serum-starved for 24 h after infection with Ad-GFP or Ad-Nrf2 and then stimulated with TGF-β for 1 h before harvesting for Western blot analysis. Quantitative analysis of p-Smad3 to total Smad3 ratio. Data in bar graph are the mean ± SE of three independent measurements. (A) ^*^ *P*<0.01 vs. control, ^**^ *P*<0.05 vs. TGF-β stimulation. (B) ^*^ *P*<0.00*1* vs. control, ^**^ *P*<0.05, ^#^ *P*<0.01 vs. TGF-β stimulation. (C) Representative semi-quantitative RT-PCR analysis of the effect of Nrf2-siRNA on DMF-induced Nrf2 mRNA expression. AD-293 cells were transfected with a control scrambled siRNA (Control-siRNA) or Nrf2-siRNA and incubated for 24 h. Cells were incubated in serum-free media for 12 h, treated with DMF (40 µmol/l) for 1 h, and harvested for semi-quantitative RT-PCR. (D) The effect of Nrf2-siRNA on DMF-induced suppression of TGF-β-stimulated (CAGA)_9_MLP-Luc activity. AD-293 cells were serum-starved for 12 h after cotransfection with (CAGA)_9_MLP-Luc and the Control-siRNA or Nrf2-siRNA for 24 h. Cells were then stimulated with TGF-β for 5 h after pretreatment with DMF (40 µmol/l) for 1 h. Data represent the means ±SEM of three independent measurements. *P<0.01 vs. reporter alone, ^**^ *P*<0.05 vs. TGF-β stimulation, and ^***^ *P*<0.05 vs. Control-siRNA with DMF treatment. (E) Representative semi-quantitative RT-PCR analysis of the effect of Nrf2-siRNA on DMF-induced inhibition on TGF-β-stimulated type I collagen mRNA expression. NRK-49F cells were transfected with Control-siRNA or Nrf2-siRNA for 24 h, and then starved for 12 h. Cells were pretreated with DMF (10 µmol/l) for 1 h, stimulated with TGF-β for 24 h, and then harvested for semi-quantitative RT-PCR analysis.

To determine whether Nrf2 inhibits TGF-β/Smad signaling, the effect of Ad-Nrf2 on TGF-β-stimulated Smad3 phosphorylation was investigated. The results revealed that Ad-Nrf2 inhibited TGF-β-stimulated Smad3 phosphorylation but had no effect on total Smad3 and Smad4 protein expression in NRK-49F cells (Figure 4A) and RMC cells (Figure 4B). To confirm further that the suppression of TGF-β/Smad3 activity and ECM protein expression by DMF is mediated by Nrf2, endogenous Nrf2 expression was down-regulated by transfecting AD-293 cells with a small interfering RNA (siRNA) against Nrf2 (Nrf2-siRNA). The Nrf2-siRNA successfully inhibited the expression of Nrf2 (Figure 4C) and significantly blocked the DMF-induced suppression of TGF-β-stimulated (CAGA)_9_MLP-Luc promoter activity (Figure 4D). Moreover, the Nrf2-siRNA reversed the inhibitory effects of DMF on TGF-β-stimulated type 1 collagen expression (Figure 4E). These data suggest that Nrf2 mediates the inhibitory effect of DMF on TGF-β/Smad signaling pathway and TGF-β-stimulated ECM protein expression.

### Anti-fibrotic activity and Inhibitory effect of DMF on the TGF-β/Smad signaling are independent of induction of ARE-driven Nrf2 target genes

**Figure 5:**
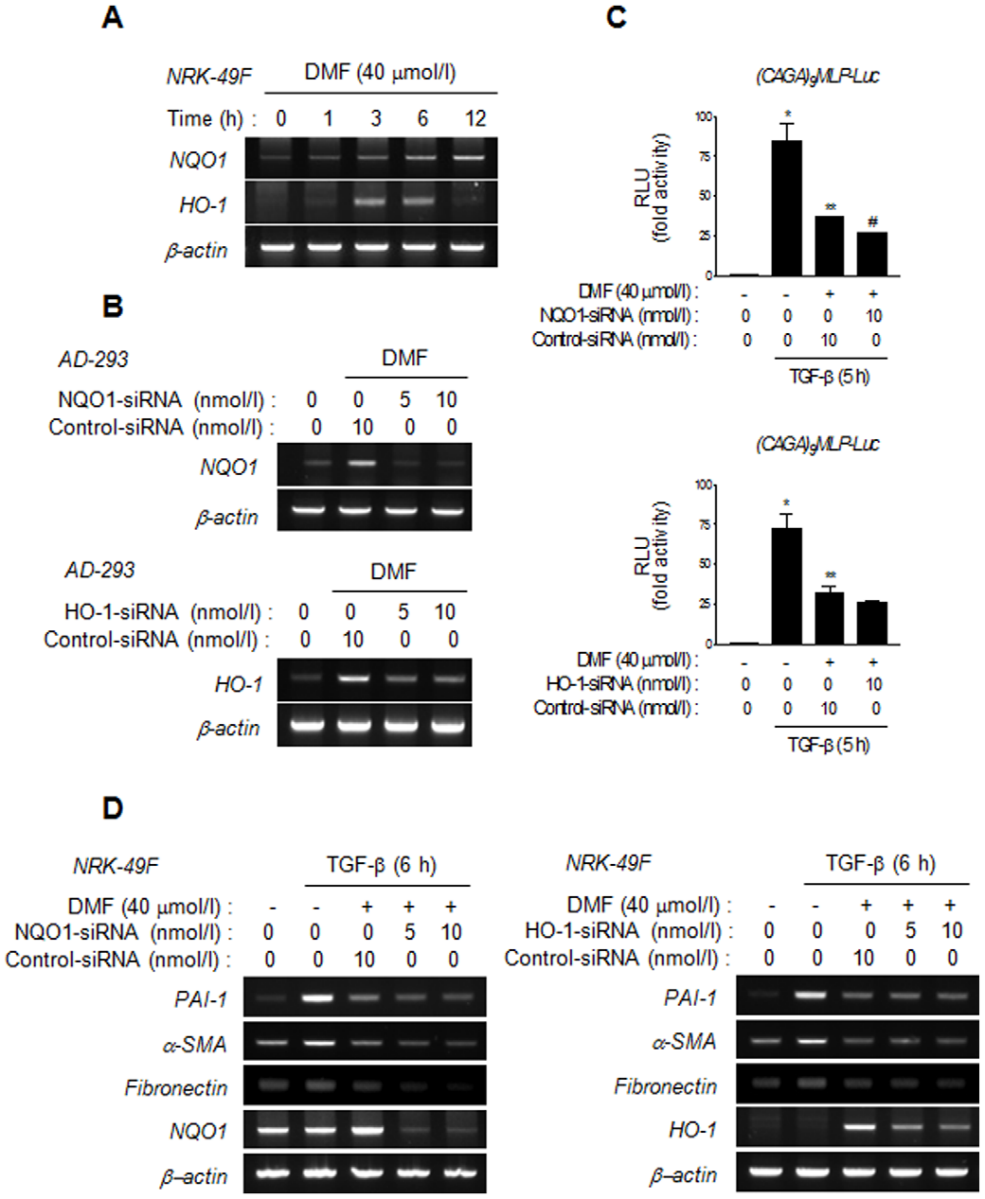
NQO1 and HO-1 antioxidant enzymes are not necessary for DMF inhibition of the TGF-β/Smad signaling pathway. (A) Representative semi-quantitative RT-PCR analysis of the effect of DMF on NQO1 and HO-1 mRNA expression in NRK-49 cells. Cells were treated with DMF (40 µmol/l) for the times indicated and then were harvested for semi-quantitative RT-PCR analysis. (B) Representative semi-quantitative RT-PCR analysis of the effect of siRNA on NQO1 and HO-1 mRNA expression in AD-293 cells. Transfected cells were incubated for 24 h, and serum starved for 12 h. Cells were treated with DMF (40 µmol/l) for 6 h and harvested. (C) The effect of downregulation of NQO1 and HO-1 on DMF-induced inhibition of TGF-β-stimulated (CAGA)_9_MLP-luciferase activity. AD-293 cells were transiently transfected with (CAGA)_9_MLP-luc reporter constructs and siRNAs against NQO1 or HO-1 for 24 h and then were serum starved for 12 h. Cells were pretreated with DMF (40 µmol/l) for 1 h and stimulated with TGF-β (2 ng/ml) for 5 h. Data are the mean ±SEM of three independent measurements. ^*^ *P*<0.05 vs. reporter alone, ^**^ *P*<0.05, vs. TGF-β stimulation and ^#^ *P*<0.05 vs. TGF-β stimulation with DMF treatment (upper panel); *P<0.05 vs. reporter alone, and ^**^ *P*<0.05 vs. TGF-β stimulation (lower panel). (D) Representative semi-quantitative RT-PCR showing the effects of NQO1 or HO-1 knock-down on DMF-mediated suppression of TGF-β-stimulated mRNA expression of PAI-1, α-SMA, and fibronectin in NRK-49 cells. NRK-49F cells were transfected with siRNAs against NQO1 or HO-1 for 24 h. Serum-starved cells were pretreated with DMF (40 µmol/l) for 1 h and stimulated with TGF-β (2 ng/ml) for 6 h.

A growing body of evidence indicates that reactive oxygen species (ROS) mediate TGF-β-induced renal fibrosis, while Nrf2 target genes, such as NQO1 and HO-1, prevent ROS-induced renal fibrosis [29]–[31]. As expected, DMF increased NQO1 and HO-1 mRNA expression in NRK-49F cells (Figure 5A). Thus, we aimed to investigate the involvement of these antioxidant enzymes in the inhibition of TGF-β/Smad signaling and TGF-β-stimulated ECM protein expression by DMF. To determine whether the induction of NQO1 and HO-1 is required for the suppressive effect of DMF on the TGF-β/Smad signaling pathway, DMF-induced NQO-1 and HO-1 expression was knocked down by siRNAs against NQO-1 (NQO1-siRNA) or HO-1 (HO-1-siRNA) (Figure 5B). However, neither NQO1 nor HO-1 siRNAs abolished the inhibitory effects of DMF on the TGF-β-stimulated (CAGA)_9_MLP-Luc promoter activity and ECM mRNA expression (Figure 5C and 5D). In a good agreement with knock-down experiments, ES936 or SnPP, chemical inhibitors of NQO1 or HO-1, respectively did not reverse the suppressive effects of DMF on TGF-β/Smad signaling and ECM expression (Figure S4). Furthermore, down-regulation of glutathione S-transferase (GST), one of other antioxidant response element (ARE)-dependent Nrf2 target genes using siRNA did not block the inhibition of TGF-β-stimulated expression of profibrotic genes by DMF (Figure S5). Collectively, these data indicated that the effects of DMF on TGF-β-stimulated Smad signaling and expression of profibrotic genes may not be mediated by the induction of ARE-driven Nrf2 target genes.

### DMF ameliorates UUO-induced renal fibrosis

Finally, an experimental UUO-induced renal fibrosis model was employed to determine whether DMF attenuates renal fibrosis *in vivo*. As expected, the architecture of the tubules was dramatically altered in UUO kidneys, resulting in the development of interstitial fibrosis.

**Figure 6:**
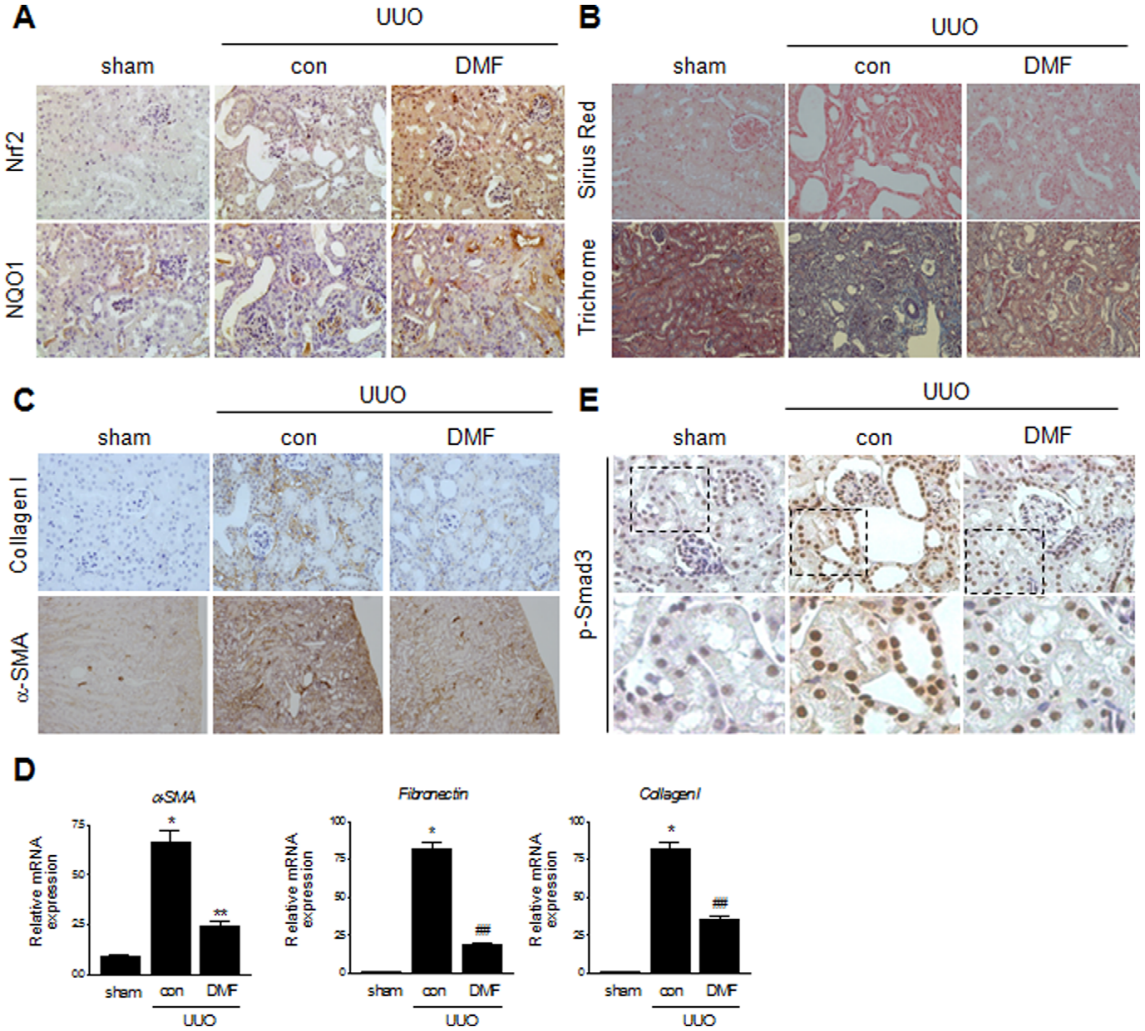
DMF attenuates UUO-induced renal fibrosis in mice. (A) Representative immunohistochemical staining for Nrf2 and NQO1. (B) Representative images of Sirius Red and trichrome staining of kidneys harvested from mice killed seven days after UUO surgery with or without DMF treatment (25 mg/kg/day). (C) Representative images of immunohistochemical staining for type I collagen and α-SMA. (D) Representative real-time PCR analysis of α-SMA, fibronectin and type 1 collagen mRNA expression in kidneys from untreated mice or mice treated with DMF. All mice were sacrificed seven days after UUO surgery. Data represent the mean ±SEM of three independent measurements (n = 5 in each group). ^*^ *P*<0.001 vs. sham control; ^**^ *P*<0.01, ^##^ *P*<0.001 vs. UUO. (E) Representative immunohistochemical staining for p-Smad3 in kidneys of mice treated with DMF (25 mg/kg/day) for seven days after UUO surgery.

Expression of Nrf2 and NQO1 was markedly increased in the renal tubules and interstitial area in the DMF-treated UUO kidneys, compared with those of sham-operated or UUO control mice (Figure 6A). Sirius Red and trichrome staining showed, in comparison with UUO control kidneys, that UUO kidneys of DMF-treated mice exhibited a marked decrease in renal fibrosis seven days after UUO (Figure 6B). Immunohistochemical staining for type 1 collagen and α-SMA revealed that renal fibrosis in UUO kidneys of DMF-treated mice was less extensive than that in UUO control kidneys (Figure 6C). Quantitative real-time PCR results showed that UUO markedly increased mRNA levels of α-SMA, fibronectin and type 1 collagen. In contrast, the UUO kidneys of DMF-treated mice showed a significant decrease in expression of all three ECM proteins analyzed (Figure 6D). Moreover, DMF markedly reduced the UUO-induced nuclear staining of phosphorylated Smad3 in the renal tubular epithelial cells (Figure 6E). Collectively, these results indicated that DMF prevents the progression of UUO-induced renal fibrosis in mice.

## Discussion

This study demonstrated that DMF effectively inhibited TGF-β-stimulated profibrotic genes and ECM protein expression in two cultured rat renal cell lines. In addition, DMF inhibited TGF-β-stimulated Smad3 phosphorylation and attenuated profibrotic gene and ECM protein expression in UUO-induced kidneys. Furthermore, our results demonstrated that Nrf2 mediated the suppressive effects of DMF on TGF-β-stimulated profibrotic genes and ECM protein expression via an ARE-independent mechanism. Taken together, our data suggest the possibility that DMF could be used for the treatment of renal fibrosis.

As mentioned previously, TGF-β expression is increased in a variety of renal diseases including obstructive nephropathy, and it has been implicated as a major mediator of ECM protein accumulation in diabetic nephropathy and tubulointerstitial fibrosis [9], [35], [36]. TGF-β phosphorylates Smad2/3, which then translocates into the nucleus where it induces the expression of PAI-1 and ECM proteins such as fibronectin and type 1 collagen. Therefore, the suppression of TGF-β signaling has been included in several therapeutic approaches for preventing renal fibrosis [37], [38]. In this study, we found that DMF attenuated TGF-β-stimulated profibrotic gene expression via the inhibition of TGF-β-induced Smad3 phosphorylation. Moreover, DMF inhibited UUO-induced ECM accumulation *in vivo*.

It is well known that DMF increases the levels of active Nrf2 [17], [23], [24]. Our previous study showed that sulforaphane-induced Nrf2 activation effectively inhibited hepatic fibrosis via the inhibition of TGF-β/Smad signaling. These data prompted us to examine whether Nrf2 mediates DMF-induced suppression of profibrotic genes and ECM protein expression in cultured renal cells induced by TGF-β and, as expected, DMF increased Nrf2 protein expression levels in cultured renal cells. Adenovirus-mediated overexpression of Nrf2 successfully inhibited TGF-β-stimulated profibrotic gene expression by inhibiting the TGF-β/Smad signaling pathway. Moreover, knockdown of Nrf2 using an siRNA reversed the inhibitory effect of DMF on TGF-β/Smad3 signaling as well as on the profibrotic genes and ECM protein expression. Our previous study demonstrated that Nrf2 interacted physically with the Smad3 protein, and repressed p-Smad3 and the trapping of p-Smad3, in cultured hepatocytes [33]. Consistent with these results, interaction between Nrf2 and Smad3 was confirmed by co-immunoprecipitaiton assay in cultured AD-293 cells (Figure S6). Additionally, DMF and Ad-Nrf2 inhibited ALK5-stimulated Smad3/4 reporter activity and Smad3 phosphorylation in cultured renal cell lines, implying that Nrf2 negatively regulates signaling molecules downstream of the TGF-β receptor. Taken together, these data suggested that DMF-induced Nrf2 might repress TGF-β-stimulated profibrotic gene and ECM protein expression via direct physical interaction with Smad3.

In recent reports, p62 interacts with the Nrf2-binding site on Keap1 and increased p62 competes with Nrf2 for the interaction with Keap1, resulting in stabilization of Nrf2 followed by transcriptional induction of ARE target genes [34]. In the present study, we found that DMF augmented p62 expression, but this increase in p62 expression by DMF occurred much later than that of Nrf2. Moreover, down-regulation of p62 expression did not affect DMF-induced Nrf2 expression, as well as repression of the TGF-β-stimulated (CAGA)_9_MLP-Luc activity and profibrotic gene expression by DMF. Taken together, Nrf2 activation by DMF is independent of p62 expression.

Increasing evidence indicates that, in addition to the TGF-β-stimulated Smad pathway, other signaling pathways, such as ROS-induced redox sensitive transcription factor pathways, are also important in the initiation and progression of renal disease [39]. It is known that TGF-β induces ROS production, which mediates profibrotic responses through Smad-independent pathways [40], [41], and thus the antioxidant activities of DMF are likely to act as potential antifibrotic agents. The antioxidant property of DMF functions via the Nrf2-dependent stimulation of antioxidant enzymes such as NQO1 and HO-1 [42] whose induction is reported to prevent the progression of fibrosis and to reverse established renal scarring in UUO rats [43]. On the basis of these data, we examined whether the DMF-induced upregulation of NQO1 and HO-1 was related to the DMF-induced suppression of profibrotic gene expression. However, intriguingly, the inhibition of ARE-driven Nrf2 target genes such as NQO1, HO-1 and GST did not reverse the DMF-induced suppression of the TGF-β/Smad signaling pathway and profibrotic gene expression, indicating that induction of ARE-driven Nrf2 target genes are not involved in mediating the effects of DMF.

In conclusion, our data demonstrated that the upregulation of Nrf2 by DMF attenuated renal fibrosis via ARE-independent inhibition of the TGF-β/Smad signaling pathway. This study suggests that pharmacologic agents, such as DMF, may provide promising prospects for overcoming the doubtful efficacy and safety of antioxidant or immunomodulatory agents currently used to treat renal fibrosis.

## Materials and Methods

### Reagents

Dimethylfumarate (DMF) was purchased from Sigma-Aldrich (St. Louis, MO). As previously described [33], recombinant human TGF-β1 and tin protoporphyrin IX (SnPP) were purchased from R&D Systems (Minneapolis, MN) and Frontier Scientific, Inc. (Logan, UT), respectively. ES936 was kindly donated by Mazence, Inc. (Suwon, Korea).

### Cell culture

NRK-49F, a normal rat kidney fibroblast cell line, and RMC, a rat kidney mesangial cell line immortalized with the plasmid pSV3-Neo were purchased from the American Type Culture Collection (Manassas, VA). AD-293, a derivative of the commonly used HEK293 human embryonic kidney cell line, was purchased from Stratagene (La Jolla, CA). NRK-49F cells were cultured in Dulbecco's Modified Eagle's Medium (DMEM; Invitrogen, Carlsbad, CA) supplemented with 5% fetal bovine serum (FBS; Hyclone, Logan, UT) and antibiotics (Gibco, Grand Island, NY). RMC cells were cultured in DMEM (Invitrogen) supplemented with 15% FBS (Hyclone) and 0.4 mg/ml G418 (Gibco). AD-293 cells were cultured in DMEM (Invitrogen) supplemented with 10% FBS and antibiotics (Gibco).

### Plasmids and promoter assay

As described previously [33], the reporter plasmids, (CAGA)_9_MLP-Luc and PAI-1-Luc (−850 to +20) were kindly donated by Drs. Jean-Michel Gauthier (Laboratoire GlaxoWellcome, France) and Bernd R. Binder (University of Vienna, Austria), respectively. The mammalian expression plasmids pcDNA3HA-ALK5TD, which encoded the constitutively active activin-like receptor kinase 5 (ALK5), and pcDNA3-Nrf2 were kind gifts from Drs. Carl-Henrik Heldin (Ludwig Institute for Cancer Research, Sweden) and Mi-Kyoung Kwak (Yeungnam University, Korea), respectively. For the promoter assay, AD-293 cells were seeded on 24-well plates, incubated for 24 h, and transfected with 300 ng/well of reporter plasmids and mammalian expression plasmids encoding Nrf2 (100 or 300 ng/well) or ALK5 (50 ng/well) using the TransIT-LT1 transfection reagent (Mirus Bio Incorporation, Madison, WI). AD-293 cells were cotransfected with (CAGA)_9_MLP-Luc and 50 nmol/l human Nrf2-siRNA, 10 nmol/l human NQO1-siRNA, 10 nmol/l human HO-1-siRNA and control siRNA duplexes (Table 1, Bioneer Corporation, Daejeon, Korea) using both TransIT-LT1 and TransIT-TKO transfection reagents (Mirus Bio Incorporation). Cytomegalovirus (CMV)-β-galactosidase plasmids were cotransfected as an internal control. Luciferase activity was normalized to that of β-galactosidase.

**Table 1:** siRNA sequences.

Gene	Sequences (5′→3′)
human Nrf2-siRNA	sense-GCUUUUGGCGCAGACAUUCdTdT
	antisense-GAAUGUCUGCGCCAAAAGCdTdG
human NQO1-siRNA	sense-CAGUACACAGAUACCUUGAdTdT
	antisense-UCAAGGUAUCUGUGUACUGdTdT
human HO-1-siRNA	sense-CCUGAGUUUCAAGUAUCCUdTdT
	antisense-AGGAUACUUGAAACUCAGGdTdT
control-siRNA	sense-CCUACGCCACCAAUUUCGUdTdT
	antisense-ACGAAAUUGGUGGCGUAGGdTdT
rat Nrf2-siRNA	sense-CUGUUGAUGACUUCAAUGAdTdT
	antisense-UCAUUGAAGUCAUCAACAGdTdT
rat NQO1-siRNA	sense-CUCUUCUCUGCCUUGUACAdTdT
	antisense-UGUACAAGGCAGAGAAGAGdTdT

### Preparation of recombinant adenovirus

A full length mouse Nrf2 cDNA was inserted into the *KpnI* and *XhoI* sites of the pAdTrack-CMV shuttle vector. The recombinant adenoviral plasmid was generated as described previously [44], and recombinant adenoviruses were amplified in HEK-293 cells and subsequently purified.

### Transfection of siRNAs, RNA isolation and RT-PCR

For siRNA transfection, 10 nmol/l rat Nrf2-siRNA, 10 nmol/l rat NQO1-siRNA, 10 nmol/l rat HO-1-siRNA and control siRNA duplexes were purchased from the Bioneer Corporation (Table 1). Cells were seeded onto 60 mm plates and simultaneously transfected with Lipofectamine™ RNAiMax reagent (Invitrogen). After incubation for 24 h, cells were starved for 12 h, and then pretreated with DMF for 1 h, cells were stimulated with TGF-β. Total RNA was extracted using Trizol reagent (Invitrogen) according to the manufacturer's instructions, and semi-quantitative RT-PCR analysis was performed as described previously [44]. An aliquot (1 µg) of total RNA was reverse transcribed using the First Strand cDNA synthesis kit (Fermentas, EU) according to the manufacturer's protocol. The first strand cDNAs were amplified by PCR using gene-specific primers [33] to determine mRNA expression levels. Quantitative real-time PCR (qRT-PCR) was carried out using Power SYBR Green PCR Master Mix (Applied Biosystems, Warrington, UK) with the StepOnePlus Real-Time PCR System (Applied Biosystems). The expression levels of β-actin (semi-quantitative RT-PCR) and GAPDH (qRT-PCR) were used as internal controls.

### Western blot analysis

Western blot analysis was performed as described previously [44] using specific primary antibodies [anti-p-Smad3 (Ser423/425); anti-Smad3 and anti-Smad4 (Cell Signaling, Beverly, MA); anti-type I collagen; anti-α-SMA and anti-β-actin (Abcam, Cambridge, MA); anti-Nrf2 (Santa Cruz Biotechnologies, Santa Cruz, CA); and anti-PAI-1 and anti-fibronectin (BD Biosciences, San Jose, CA). For Western blot analysis of type I collagen, cell lysates were subjected to native polyacrylamide gel electrophoresis (native PAGE: non-heating and non-denaturing conditions). Quantitative analysis of p-Smad3 to total Smad3 ratio was conducted with NIH Image J software.

### Ethics statement

All experimental procedures were carried out in accordance with the appropriate institutional guidelines for animal research. The protocol was approved by the Committee on the Ethics of Animal Experiments of the Keimyung University School of Medicine (Permit Number: KM-2010–29).

### Mouse UUO-induced renal fibrosis model

Eight-week-old male C57BL/6 mice (Hyochang Science, Daegu, Korea) were divided into three groups (sham-operated control, UUO mice and UUO mice treated with 25 mg/kg of DMF, n = 5/group) for each experimental regime. For UUO surgery, the animals were anesthetized with pentobarbital (50 mg/kg) and the left ureter was doubly ligated. Sham animals underwent the same surgical procedures except for ligation. Following UUO, mice in the DMF-treated group were dosed with DMF (25 mg/kg/day) by gavage daily for seven days. All mice were sacrificed seven days after UUO surgery.

### Immunohistochemical analysis

Kidney tissues were fixed with 4% paraformaldehyde (PFA, Sigma-Aldrich) and paraffin embedded. Kidney sections (4 mm) were deparaffinized in xylene, rehydrated through descending grades of ethanol and subjected to immunohistochemical analysis using antibodies against Nrf2, NQO1 (Santa Cruz), type I collagen, α-SMA and p-Smad3 [33], [45]. To evaluate renal fibrosis, sections were stained with Sirius Red (Sigma-Aldrich) and trichrome (Sigma-Aldrich) according to the manufacturer's instructions.

### Statistical analysis

Data are expressed as means ±SEM. Statistical analyses were performed using an unpaired Student's t-test and a value of *P*<0.05 was considered statistically significant.

## Supporting Information

Figure S1 **DMF-induced Nrf2 expression is sustained up to 24 h in the presence of TGF-β.** NRK-49F cells were serum starved for 12 h and pretreated with DMF (40 µmol/l) for 1 h. Cells were stimulated with TGF-β (2 ng/ml) for 24 h and then harvested for Western blot analysis.
(TIF)

Figure S2 **DMF increases nuclear Nrf2 expression.** Serum-starved RMC cells were treated with indicated doses of DMF for 1 h and harvested. Nuclear and cytosolic fractions were isolated for Western blot analysis. Lamin B and β-tubulin were used as nuclear and cytosolic markers, respectively. Quantitative analysis of Nrf2/Lamin B ratio was conducted with NIH Image J software. Data are the mean ±SEM of three independent measurements. *P<0.001, **P<0.01 vs. control.
(TIF)

Figure S3 **Effects of DMF-induced p62 on the Nrf2 expression and TGF-β-stimulated upregulation of porfibrotic genes.** (A) Representative western blot analysis of p62 expression in DMF-treated NRK-49F cells. Cells were serum starved for 12 h and treated with DMF (40 µmol/L) for indicated times. (B) Representative Western blot analysis showing the effect of p62 knock-down on DMF-induced Nrf2 expression. NRK-49F cells were transfected with p62-siRNA for 24 h and serum starved for 12 h. (C, D) The effect of knock-down of p62 on (CAGA)_9_MLP-Luc activity stimulated by TGF-β. NRK-49F cells were seed on 24-well plate and incubated for 24 h. Cells were transfected with a (CAGA)_9_MLP-luc reporter construct for 24 h and then serum starved for 12 h. DMF was treated for 1 h and stimulated with TGF-β (2 ng/ml) for 5 h. *P<0.01 vs. reporter alone, and ^**^ *P*<0.05 vs. TGF-β stimulation. (E) Representative western blot analysis showing the effect of knock-down of p62 on DMF-induced suppression of TGF-β-stimulated PAI-1 and α-SMA protein expression. NRK-49F cells were transfected with p62-siRNA for 24 h and then serum starved for 12 h. TGF-β (2 ng/ml) was treated for 12 h after treatment of DMF for 1 h.
(TIF)

Figure S4 **NQO1 and HO-1 antioxidant enzymes are not necessary for DMF-induced inhibition of the TGF-β/Smad signaling pathway.** (A) The effects of ES936 and SnPP on DMF-induced suppression of (CAGA)_9_MLP-luciferase activity stimulated by TGF-β. AD-293 cells were transfected with a (CAGA)_9_MLP-luc reporter construct for 24 h and then were serum starved for 12 h. Cells were pretreated with DMF (40 µmol/l) and either ES936 (upper panel) or SnPP (lower panel), inhibitors of NQO1 and HO-1, respectively. After 1 h, cells were stimulated with TGF-β (2 ng/ml) for 5 h. Data are the mean ±SEM of three independent measurements. *P<0.001 vs. reporter alone, ^**^ *P*<0.001 vs. TGF-β stimulation and ^#^ *P*<0.01, ^**^ *P*<0.001 vs. TGF-β stimulation with DMF treatment (upper panel); *P<0.01 vs. reporter alone, and ^**^ *P*<0.05 vs. TGF-β stimulation (lower panel). (B and C) The effects of ES936 and SnPP on DMF-induced suppression of TGF-β-stimulated mRNA expression of PAI-1, α-SMA, fibronectin and type I collagen in NRK-49 cells. Serum starved cells were pretreated with DMF and ES936 or SnPP. After 1 h, cells were stimulated with TGF-β (2 ng/ml) for 6 h (B) or 24 h (C) and then harvested for semi-quantitative RT-PCR analysis. (D) Representative Western blot analysis showing the effects of ES936 and SnPP on DMF-induced suppression of TGF-β-stimulated PAI-1 and α-SMA protein expression. After serum starvation for 12 h, NRK-49F cells were pretreated with DMF and ES936 or SnPP for 1 h. Cells were stimulated with TGF-β (2 ng/ml) for 12 h and harvested for Western blot analysis.
(TIF)

Figure S5 **GST antioxidant enzyme is not necessary for DMF-mediated inhibition of the TGF-β/Smad signaling pathway.** NRK-49F cells were transfected with indicated doses of rat GST-siRNA (Gsta3) for 24 h, and treated with DMF (40 µmol/l) for 1 h. Cells were stimulated with TGF-β (2 ng/ml) for 6 h and harvested for semi-quantitative RT-PCR.
(TIF)

Figure S6 **Nrf2 interacts with Smad3.** AD-293 cells were transiently transfected with pcDNA3-Smad3 construct for 36 h, and then harvested for immunnoprecipitaion. Cell lysate was precipitated with anti-Smad3 antibody for 12 h, and subjected to Western blotting with anti-Nrf2 antibody.
(TIF)

Materials and Methods S1 **Supplemental methods.**
(DOCX)
