# Correction: Dimethylfumarate Attenuates Renal Fibrosis via NF-E2-Related Factor 2-Mediated Inhibition of Transforming Growth Factor-β/Smad Signaling

**DOI:** 10.1371/annotation/a1a8aba9-4ae9-464e-83e9-f2d8a6f3f890

**Published:** 2013-09-09

**Authors:** Chang Joo Oh, Joon-Young Kim, Young-Keun Choi, Han-Jong Kim, Ji-Yun Jeong, Kwi-Hyun Bae, Keun-Gyu Park, In-Kyu Lee

The number of the third-listed grant from the National Research Foundation is incorrect. The first sentence of the Funding Statement should read: "This work was supported by grants from the National Research Foundation (2011-0028659; WCU Program, R32-10064; Future-based Technology Development Program Bio Field, 2010-0019514; and Basic Science Research Program, 2012-0006798 to J-YK and 2012-0001350 to H-JK), funded by the Korean Ministry of Education, Science and Technology." 

