# Supplementary figures and images for: Dimethylfumarate Attenuates Renal Fibrosis via NF-E2-Related Factor 2-Mediated Inhibition of Transforming Growth Factor-β/Smad Signaling
Source: PLoS One. 2012 Oct 8;7(10):e45870. doi: 10.1371/journal.pone.0045870 (PMC3466265; doi:10.1371/journal.pone.0045870)

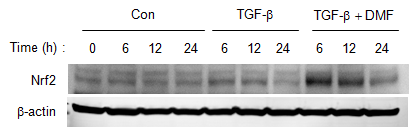

Supplement: Figure S1 — DMF-induced Nrf2 expression is sustained up to 24 h in the presence of TGF-β. NRK-49F cells were serum starved for 12 h and pretreated with DMF (40 µmol/l) for 1 h. Cells were stimulated with TGF-β (2 ng/ml) for 24 h and then harvested for Western blot analysis. (TIF) [file pone.0045870.s001.tif]

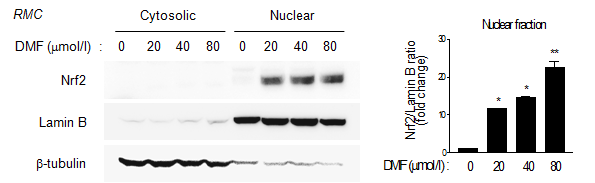

Supplement: Figure S2 — DMF increases nuclear Nrf2 expression. Serum-starved RMC cells were treated with indicated doses of DMF for 1 h and harvested. Nuclear and cytosolic fractions were isolated for Western blot analysis. Lamin B and β-tubulin were used as nuclear and cytosolic markers, respectively. Quantitative analysis of Nrf2/Lamin B ratio was conducted with NIH Image J software. Data are the mean ±SEM of three independent measurements. *P<0.001, **P<0.01 vs. control. (TIF) [file pone.0045870.s002.tif]

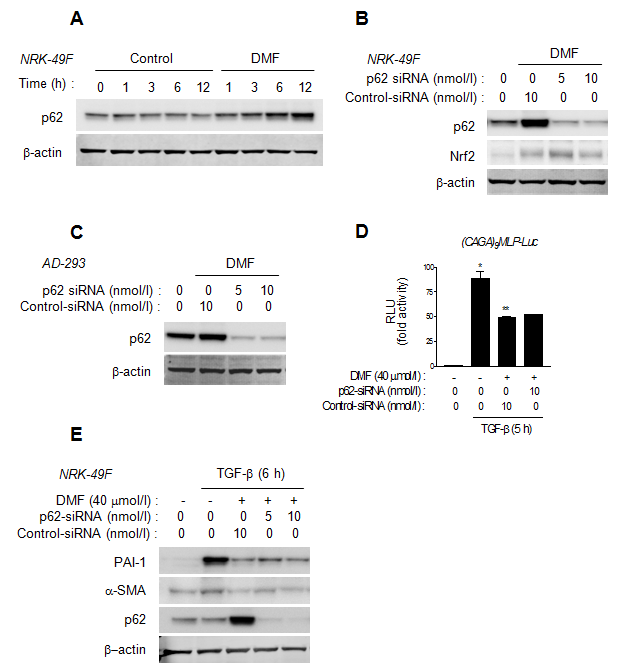

Supplement: Figure S3 — Effects of DMF-induced p62 on the Nrf2 expression and TGF-β-stimulated upregulation of porfibrotic genes. (A) Representative western blot analysis of p62 expression in DMF-treated NRK-49F cells. Cells were serum starved for 12 h and treated with DMF (40 µmol/L) for indicated times. (B) Representative Western blot analysis showing the effect of p62 knock-down on DMF-induced Nrf2 expression. NRK-49F cells were transfected with p62-siRNA for 24 h and serum starved for 12 h. (C, D) The effect of knock-down of p62 on (CAGA)9MLP-Luc activity stimulated by TGF-β. NRK-49F cells were seed on 24-well plate and incubated for 24 h. Cells were transfected with a (CAGA)9MLP-luc reporter construct for 24 h and then serum starved for 12 h. DMF was treated for 1 h and stimulated with TGF-β (2 ng/ml) for 5 h. *P<0.01 vs. reporter alone, and ** P<0.05 vs. TGF-β stimulation. (E) Representative western blot analysis showing the effect of knock-down of p62 on DMF-induced suppression of TGF-β-stimulated PAI-1 and α-SMA protein expression. NRK-49F cells were transfected with p62-siRNA for 24 h and then serum starved for 12 h. TGF-β (2 ng/ml) was treated for 12 h after treatment of DMF for 1 h. (TIF) [file pone.0045870.s003.tif]

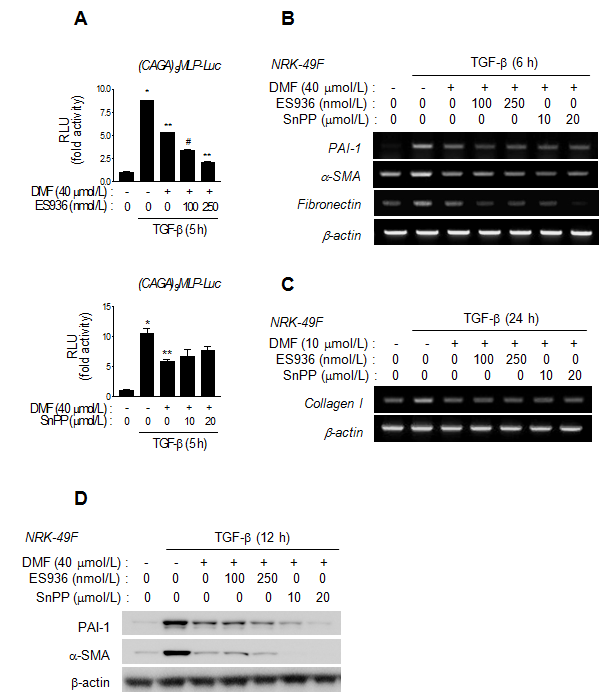

Supplement: Figure S4 — NQO1 and HO-1 antioxidant enzymes are not necessary for DMF-induced inhibition of the TGF-β/Smad signaling pathway. (A) The effects of ES936 and SnPP on DMF-induced suppression of (CAGA)9MLP-luciferase activity stimulated by TGF-β. AD-293 cells were transfected with a (CAGA)9MLP-luc reporter construct for 24 h and then were serum starved for 12 h. Cells were pretreated with DMF (40 µmol/l) and either ES936 (upper panel) or SnPP (lower panel), inhibitors of NQO1 and HO-1, respectively. After 1 h, cells were stimulated with TGF-β (2 ng/ml) for 5 h. Data are the mean ±SEM of three independent measurements. *P<0.001 vs. reporter alone, ** P<0.001 vs. TGF-β stimulation and # P<0.01, ** P<0.001 vs. TGF-β stimulation with DMF treatment (upper panel); *P<0.01 vs. reporter alone, and ** P<0.05 vs. TGF-β stimulation (lower panel). (B and C) The effects of ES936 and SnPP on DMF-induced suppression of TGF-β-stimulated mRNA expression of PAI-1, α-SMA, fibronectin and type I collagen in NRK-49 cells. Serum starved cells were pretreated with DMF and ES936 or SnPP. After 1 h, cells were stimulated with TGF-β (2 ng/ml) for 6 h (B) or 24 h (C) and then harvested for semi-quantitative RT-PCR analysis. (D) Representative Western blot analysis showing the effects of ES936 and SnPP on DMF-induced suppression of TGF-β-stimulated PAI-1 and α-SMA protein expression. After serum starvation for 12 h, NRK-49F cells were pretreated with DMF and ES936 or SnPP for 1 h. Cells were stimulated with TGF-β (2 ng/ml) for 12 h and harvested for Western blot analysis. (TIF) [file pone.0045870.s004.tif]

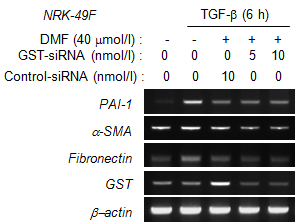

Supplement: Figure S5 — GST antioxidant enzyme is not necessary for DMF-mediated inhibition of the TGF-β/Smad signaling pathway. NRK-49F cells were transfected with indicated doses of rat GST-siRNA (Gsta3) for 24 h, and treated with DMF (40 µmol/l) for 1 h. Cells were stimulated with TGF-β (2 ng/ml) for 6 h and harvested for semi-quantitative RT-PCR. (TIF) [file pone.0045870.s005.tif]

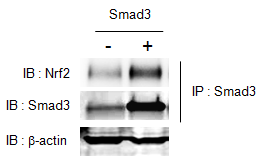

Supplement: Figure S6 — Nrf2 interacts with Smad3. AD-293 cells were transiently transfected with pcDNA3-Smad3 construct for 36 h, and then harvested for immunnoprecipitaion. Cell lysate was precipitated with anti-Smad3 antibody for 12 h, and subjected to Western blotting with anti-Nrf2 antibody. (TIF) [file pone.0045870.s006.tif]
