# Supplementary material for: Dimethylfumarate Attenuates Renal Fibrosis via NF-E2-Related Factor 2-Mediated Inhibition of Transforming Growth Factor-β/Smad Signaling
Source: PLoS One. 2012 Oct 8;7(10):e45870. doi: 10.1371/journal.pone.0045870 (PMC3466265; doi:10.1371/journal.pone.0045870)
Supplement: Materials and Methods S1 — Supplemental methods. (DOCX) [file pone.0045870.s007.docx]

**Materials and Methods S1**

**Isolation of Nucleus and Cytosol**

RMC cells were pretreated with several doses of DMF for 1 h, and then stimulated with TGF-β (2 ng/ml) for 1h. Cells were harvested after trypsinization and lysed with Buffer A [10 mM HEPES (pH7.9), 10 mM KCl, 0.1 mM EDTA, 1 mM DTT and 0.5 mM PMSF]. After adding 10% NP-40, cells were centrifuged and supernatant (cytosol fraction) was collected. Again, cell pellet was lysed with Buffer B [20 mM HEPES (pH7.9), 0.4 M NaCl, 1 mM EDTA, 1 mM DTT and 1 mM PMSF] and then centrifuged. Collected supernatant was used for nucleus fraction. Cell extract was loaded on SDS-PAGE and detected with ECL solution. Lamin B and β-tubulin were used as nuclear and cytosolic markers, respectively. Quantitative analysis of Nrf2/Lamin B ratio was conducted with NIH Image J software.

**Transfection of siRNA**

AD-293 cells were seeded on 24-well plate or 60 mm dishes and transfected with different doses of human p62-siRNA (sense-GUGAACUCCAGUCCCUACAdTdT; antisense-UGUAGGGACUGGAGUUCACdTdT) for 24 h using RNAiMAX reagent. Cells were starved for 12 h, and then stimulated with TGF-β (2 ng/ml) for 5~6 h. Cells were lysed for Western blot or (CAGA)_9_MLP-Luc promoter activity. Also, NRK-49F cells were seeded on 100 mm dishes and transfeced with diverse doses of rat p62-siRNA (sense- CAUUUGUGAUGGUUGCAAUTdT; antisense-AUUGCAACCAUCACAAAUCdTdT) or GST-siRNA (Gsta3, sense-CACUUCCUCAGAUAUUACUdTdT; antisense AGUAAUAUCUGAGG AAGUGdTdT) for 24 h and serum starved for 12 h. Cells were stimulated with TGF-β (2 ng/ml) for 6 h and then lysed for Western blot.

**Co-immunoprecipitation assay**

AD-293 cells were transfected with pcDNA-Smad3 construct for 36 h. Cells were lysed with lysis buffer [50 mM Tris-HCl (pH 7.4), 150 mM NaCl, 1 mM EDTA, 1% Triton X-100, 50 mM NAF, 1 mM Na_3_VO_4_, 10 mM Na_4_P_2_O_7_, 7 μg/ml Aprotinin, 7 μg/ml Leupeptin, 100 μM PMSF and phospatase inhibitor cocktail (Sigma-Aldrich)]. Cell extract was incubated with Proteins G PLUS-Agarose (Santa Cruz) and then incubated with Smad3 antibody for 16 h. Agarose beads were washed with cold-PBS and cell lysate was loaded on SDS-PAGE.
